# Association between urinary tract infections and anemia among the elderly in India: Insights from the longitudinal aging study

**DOI:** 10.1371/journal.pgph.0004390

**Published:** 2025-06-18

**Authors:** Dilwar Hussain, Jenica Barnwal

**Affiliations:** 1 PhD Scholar, Centre for the Study of Regional Development, School of Social Sciences, Jawaharlal Nehru University, New Delhi, India; 2 PhD Scholar, School of Health Systems Studies, Tata Institute of Social Sciences, Mumbai, Maharashtra, India; PLOS: Public Library of Science, UNITED STATES OF AMERICA

## Abstract

Urinary tract infections (UTIs) among the elderly remain an overlooked and important health concern, which can hamper their physical and overall well-being. From an epidemiological perspective, UTIs represent a common yet preventable infection. This study aimed to examine the association between UTIs and anemia in India, along with other determinants, using data from the Longitudinal Aging Study in India (LASI), Wave 1 (2017–18), among the elderly (aged more than 60). It explores how health, socio-demographic, economic, and regional factors contribute to UTI prevalence, providing insights into the underlying vulnerabilities within this population. Descriptive statistics, variance inflation factor, and multivariate binary logistic regression models were applied to examine the association between UTIs and factors such as anemia, age, gender, education, income, and access to sanitation facilities. Results revealed that about 2.5% of the elderly had UTIs, with higher rates observed in males, those over 80 years old, and individuals who were diabetic or anemic. The study also sheds light upon geographical variations, with the Eastern region having the highest prevalence and Southern India the lowest. Major predictors of UTIs include advancing age, lower income, experience of stroke, diagnosis of hypertension, presence of diabetes, lack of education, and inadequate sanitation, with anemia serving as a significant risk factor. Surprisingly, higher education levels were associated with increased self-reporting of UTIs, likely due to greater awareness. Tackling UTIs and related health concerns among India’s elderly requires targeted interventions strengthening healthcare access, improving sanitation, and promoting public awareness. With a growing aging population, these measures are important to enhancing their well-being and ensuring healthier later years, leading to the achievement of SDG 3.

## Background

Urinary tract infections (UTI) are among modern lifestyles' most common health issues. They are gradually becoming a global health concern. It is caused predominantly by bacterial pathogens [1,2]. UTIs can involve various components of the urogenital system, including the kidneys, bladder, ureters, and urethra [3]. The symptoms of UTIs typically include nausea, vomiting, fatigue, dysuria (painful urination), foul-smelling urine, and dark-colored urine [4,5]. UTIs affect a substantial portion of the global population, with an estimated 150 million individuals impacted annually [6–8]. UTIs are susceptible among both sexes, but females experience a higher prevalence, with around half (50%) of women encountering a UTI at some point in their lives [9], compared to about 12% of men [10]. UTIs can be classified into uncomplicated or complicated. Uncomplicated UTIs typically occur in healthy individuals without structural or functional urinary tract abnormalities, often presenting as cystitis [11]. Complicated UTIs arise from factors that impair urinary tract function, such as renal failure, kidney transplantation, pregnancy, older age, and the presence of foreign bodies like calculi, indwelling catheters, or other drainage devices [12–14].

Previous studies suggested that UTIs can arise from various factors, including poor personal [15] and environmental hygiene, as well as iron deficiency anemia, among which the latter is one of the important determinants [16–18]. Anemia is a blood disorder characterized by insufficient red blood cells or haemoglobin to transport oxygen throughout the body, often caused by iron deficiency [19]. Iron deficiency anemia increases susceptibility to infections, with UTIs being one of the most common infections [16,20,21]. Iron deficiency anemia weakens the immune system, impairing the body’s ability to combat infections. Low iron levels reduce the effectiveness of immune cells and disrupt the protective bacteria in the urinary tract, creating a favorable environment for infection [20]. Consequently, individuals with iron deficiency anemia are more vulnerable to UTIs. However, a controversy exists regarding iron supplementation, with some studies suggesting that individuals using iron supplements may face a higher risk of developing certain infections [22,23].

The existing literature has explored UTIs from clinical [24–30] and non-clinical perspectives [9,16,18,30,31]. A case-control study among graduate women found that an active sexual life is associated with an increased risk of UTIs. In contrast, no significant association was observed between the type of clothing worn and UTI prevalence [32]. Another clinical investigation found that older men are at a higher risk of UTIs compared to younger men, primarily due to underlying urologic abnormalities that become more common with age [33]. Furthermore, studies have found that UTI is also associated with economic factors such as cost per visit to healthcare [15], socio-economic status, diabetes [16], alcohol use [17], and educational level [34]. In contrast, a study found no significant association between socio-demographic factors, such as marital status, place of residence, religion, and UTI prevalence [24].

The relationship between anemia and UTI prevalence has also been explored, particularly among pregnant women [17,18,16]. A study conducted in Iran revealed that approximately 49% of pregnant women had UTIs and also found a significant relationship between anemia and UTI occurrence [16]. These findings align with other studies that have reported similar associations between anemia and increased UTI risk in pregnant women [35,36]. Although there is evidence exploring factors associated with UTI prevalence across different age groups and sexes globally [26,27,37–39], fewer studies reveal the prevalence of UTIs among the elderly in India [29,40–42]. Additionally, while some studies have found an association between UTI prevalence and anemia among pregnant women [17,18,16,43], there is a dearth of research examining the link between anemia and UTI prevalence among the elderly in India, a group already burdened with multiple morbidities, including arthritis [44], hypertension [45], obesity [46], respiratory illnesses [47], etc.

Older adults’ biological susceptibility to UTIs, caused by age-related changes like decreased bladder emptying and impaired immune function, bolsters this study [40]. Moreover, multiple chronic conditions may complicate UTI management in this age group. Even a slight prevalence of UTIs could result in a substantial public health burden given India’s anticipated increase in the elderly population. Therefore, understanding the association between anemia and UTI prevalence in the elderly is imperative to develop targeted policies and interventions that are suited to older adults' needs. To fill these voids, the current study primarily examines the association between self-reported UTI prevalence and anemia among the old population (aged 60+) in India. Furthermore, the study will explore the risk factors associated with UTIs among the elderly population using the nationally representative dataset of the Longitudinal Aging Study in India.

## Method

### Data source

The Longitudinal Aging Study in India (LASI), Wave 1 (2017–18), is a nationally representative survey focusing on individuals aged 45 and above, with a specific emphasis on the elderly population (aged 60+).The dataset, conducted across 35 states and union territories, encompasses over 72,000 respondents, including approximately 31,000 elderly, using a multistage stratified sampling design. LASI provides rich health, socioeconomic, and demographic data, including self-reported health conditions, biomarkers (e.g., anemia), economic status, behavioral risk factors (e.g., alcohol use), and sanitation facilities. Its comprehensive health metrics and detailed socioeconomic and regional variables make it ideal for analyzing aging-related issues and their determinants. However, some health conditions, such as UTIs, are self-reported, which could introduce bias. Despite this, LASI’s scope and geographic variability enable critical insights into aging, health disparities, and the interplay of socioeconomic and demographic factors. The sample of the aged 60 and above population was 30,888, out of which we dropped 88 samples due to missing information on UTIs. Finally, the total sample of this study was 30804, and information on UTIs was available. We categorized UTIs into a binary variable; one means having UTIs, and zero means not being present.

### Dependent variable

The dependent variable in the study is the self-reported prevalence of UTIs among the elderly population aged 60 years and above. This variable is binary, categorized as “Yes =1” if the respondent reported having a UTI and “No = 0” otherwise, based on responses from the Longitudinal Aging Study in India (LASI) dataset. UTIs, a common health condition among the elderly, are influenced by various factors, including biological, socioeconomic, and environmental determinants. The study specifically examines the prevalence of UTIs in relation to anemia and other socio-demographic, economic, and health-related predictors to understand their contribution to the burden of this condition among India’s elderly.

### Primary exposure variable

The primary exposure variable in the study is anemia, defined as a binary variable indicating whether the respondent is anemic (Yes/No) based on self-reported data from the LASI. Anemia, a condition characterized by insufficient hemoglobin or red blood cells, is a critical determinant of susceptibility to infections, including UTIs, due to its adverse effects on immune function. Individuals with anemia often experience weakened immunity, impairing the body’s ability to combat infections and increasing the risk of conditions like UTIs. The study explores this association by examining how anemia influences the likelihood of UTI prevalence among the elderly population in India, highlighting its role as a significant risk factor.

### Covariates

The study included several covariates, selected based on existing literature [27,38,40,48], to examine factors associated with the prevalence of UTIs among the elderly population. Age was categorized into five groups: 60–64, 65–69, 70–74, 75–79, and 80 + years. Sex was measured as a binary variable (Male/Female). Place of residence was categorized as rural or urban, and the region was classified into south, central, east, west, north, and north-east, educational level was categorized into no schooling, primary, secondary, higher Secondary, graduate, and professional course, caste was classified into scheduled tribe, scheduled caste, other backward classes, and others, while religion was categorized as hindu, muslim, christian, and other, Annual income was divided into five groups: less than 1 lakh INR, 1–1.5 lakh INR, 1.5–2 lakh INR, 2–3 lakh INR, and more than 3 lakh INR. Health-related factors diabetes, stroke, hypertension, cancer, and alcohol consumption were included as binary variables (Yes/No), as each is a known contributor to infection risk. Lastly, sanitation was assessed through the type of toilet facility, which was categorized as flush or pour-flush toilet, pit latrine, twin pit/composting toilet, and no facility/open defecation.

### Statistical analysis

Descriptive statistics were conducted to analyze the distribution of study participants based on key predictors, covariates, and outcome variables. Bivariate percentage distributions were estimated to assess the prevalence of UTIs among the elderly population in India, with sample weights applied for percentage distribution estimation. Pearson’s chi-square tests were applied to assess associations between UTIs and selected background characteristics.

To further explore the relationship between UTIs and anemia, a series of binary logistic regression models were employed. The first model estimated the crude association between anemic status and UTIs. The second model adjusted for mediating factors, including socio-demographic health, behavioral factors, and parental outcomes. In the third model, the regional factors such as place of residence and geographical region, were incorporated, and the final model included the economic and toilet type factors of household wealth to estimate, accounting for potential mediating factors and confounders. The regression results were presented as estimated adjusted odds ratios (AOR) with 95% confidence intervals (CI), with a significance level set at p < 0.05. All statistical analyses were performed using STATA version 17.0.

## Results

### Profile of the respondents, LASI (2017–18)

The sample of the elderly population used in this study is presented in Table 1. Approximately one-third of the elderly were aged between 60–64 years, with females comprising slightly more than half (52%) of this group. At the time of the survey, nearly 53% of the elderly had no formal education. Around two-thirds resided in rural areas (66.3%), and about two-fifths reported an annual income of less than one lakh rupees (41.8%). A substantial proportion of the elderly lived in Southern India (25.8%) and predominantly practiced Hinduism (73.1%). Nearly one-third belonged to Scheduled Castes (SC) or Scheduled Tribes (ST). Approximately 4% were anemic, one-sixth were diabetic, and one-third had been diagnosed with hypertension. Additionally, 0.75% were diagnosed with cancer, and about 2.6% had experienced a stroke. About 9% reported habitual alcohol consumption, and nearly 20% either practiced open defecation or lacked access to toilet facilities.

**Table 1:** Sociodemographic characteristics of the study population, India, LASI, 2017–18.

Background characteristics	n	%
**Had UTIs**		
No	30,020	97.45
Yes	784	2.55
**Anaemic**		
Yes	1,230	3.99
No	29,574	96.01
**Age (In Years)**		
60-64	9,938	32.26
65-69	8,673	28.16
70-74	5,601	18.18
75-79	3,278	10.64
80+	3,314	10.76
**Sex**		
Male	14,793	48.02
Female	16,011	51.98
**Place Of Residence**		
Rural	20,430	66.32
Urban	10,374	33.68
**Education**		
Uneducated	16,566	53.78
Primary	7,378	23.95
Secondary	4,511	14.64
Higher Secondary	1,064	3.45
Graduate	1,056	3.43
Professional Couse	229	0.74
**Income In INR (Lakhs)**		
< 1	12,896	41.86
1-1.5	4,640	15.06
1.5-2	3,237	10.51
2-3	4,083	13.25
> 3	5,948	19.31
**Region**		
Southern	7,977	25.9
Central	4,194	13.62
Eastern	5,724	18.58
Western	3,475	11.28
Northern	5,707	18.53
North-eastern	3,727	12.1
**Caste**		
Scheduled Tribe	5,082	16.5
Scheduled Caste	5,109	16.59
OBC	11,666	37.87
Other	8,947	29.04
**Religion**		
Hindu	22,544	73.19
Muslim	3,642	11.82
Christian	3,089	10.03
Other	1,529	4.96
Ever diagnosed with stroke		
No	29,978	97.33
Yes	824	2.67
Ever diagnosed with hypertension		
No	20,046	65.07
Yes	10,758	34.93
Ever diagnosed with cancer		
No	30,569	99.25
Yes	235	0.75
**Diabetic**		
Yes	4,744	15.4
No	26,060	84.61
**Habit Of Alcohol**		
Yes	2,828	9.18
No	27,976	90.82
**Toilet Facility**		
Flush Or Pour Flush Toilet	15,818	51.35
Pit Latrine	7,554	24.52
Twin Pit/Composting Toilet	1,013	3.29
No Facility, Use Open Space	6,419	20.83

### Variations in the prevalence of urinary tract infection by background characteristics

The prevalence of UTIs among Indian elderly, categorized by their background characteristics, is presented in Table 2. Approximately 2.5% of the elderly population experienced UTIs. The highest prevalence (3.1%) was observed among the oldest individuals (aged above 80) and male elders. Elderlies residing in urban areas showed a higher prevalence of UTIs than those in rural regions. Surprisingly, elderly individuals with graduate-level education had a higher prevalence of UTIs (3.7%) than those with no formal education (2%).

**Table 2:** Determinants of UTIs among the elderlies, LASI, 2017–18.

*Background Characteristics*	*Prevalence of UTI*	*P value*
**Anaemic**		<0.001
Yes	10.83	
No	2.19	
**Age (in Years)**		<0.001
60-64	2.21	
65-69	2.41	
70-74	2.56	
75-79	3.05	
80+	3.58	
**Sex**		<0.001
Male	3.1	
Female	2.11	
**Place of Residence**		<0.001
Rural	2.51	
Urban	2.80	
**Education**		<0.001
Uneducated	2.08	
Primary	3.37	
Secondary	3.06	
Higher Secondary	2.86	
Graduate	3.72	
Professional Couse	2.34	
**Income in INR (Lakhs)**		<0.001
< 1	2.38	
1-1.5	2.95	
1.5-2	2.95	
2-3	2.87	
> 3	2.31	
**Region**		<0.001
Southern	1.56	
Central	2.94	
Eastern	3.13	
Western	2.73	
Northern	2.49	
North-eastern	2.56	
**Caste**		<0.001
Scheduled Tribe	2.0	
Scheduled Caste	2.59	
OBC	2.31	
Other	3.2	
**Religion**		<0.001
Hindu	2.6	
Muslim	2.92	
Christian	1.52	
Other	1.96	
Ever diagnosed with stroke		<0.001
No	2.53	
Yes	4.45	
Ever diagnosed with hypertension		<0.001
No	2.05	
Yes	3.7	
Ever diagnosed with cancer		0.195
No	2.59	
Yes	1.75	
**Diabetic**		<0.001
Yes	3.1	
No	2.5	
**Habit of Alcohol**		<0.001
Yes	2.64	
No	1.88	
**Toilet Facility**		<0.001
Flush Or Pour Flush Toilet	2.56	
Pit Latrine	2.87	
Twin Pit/Composting Toilet	3.18	
No Facility, Use Open Space	2.35	

The prevalence of UTIs was lowest among the elderly with an annual income exceeding 3 lakh INR or among economically well-off individuals. Conversely, the highest prevalence was observed among those in the ‘Other’ social category. The lowest prevalence was found among the elderly in South India (1.5%), while the highest prevalence was observed in the Eastern region (3.1%). Around 2.5% of the elderly who reported UTIs practised Islam and had access to flush toilets. One in every ten elderly individuals found to be anemic also reported having a UTI. Among those who reported UTIs, approximately 3.7% were diagnosed with hypertension, 1.75% with cancer, and 4.4% with stroke. Furthermore, 3.1% of elderly individuals with diabetes reported having UTIs, and 2.6% of them had a habit of consuming alcohol.

### Findings from models of logistic regression for urinary tract infections among the elderly in India

The results from the three logistic regression models are summarized in Table 3. In Model 1, the crude analysis revealed a strong association between the prevalence of urinary tract infections (UTIs) and anemia. Elderly individuals with UTIs were 5.5 times more likely to be anemic (AOR: 5.49, 95% CI: 4.52–6.67). In Model 2, after adjusting for socio-demographic and health-related factors, the likelihood of anemia among individuals with UTIs increased to 5.95 times and remained statistically significant (AOR: 5.95, 95% CI: 4.87–7.27). Model 3, which additionally controlled for regional, economic, and sanitation-related factors, sustained a significant association between UTIs and anemia (AOR: 5.87, 95% CI: 4.80-7.18).

**Table 3:** Factors influencing UTIs among the elderlies in India, LASI, 2019–20.

Predictors	Model 1	Model 2	Model 3
**Anaemic**			
No	Ref	Ref	Ref
Yes	5.49 (4.52-6.67) ***	5.95 (4.87-7.27) ***	5.87 (4.80-7.18) ***
**Age**			
60-64		Ref	Ref
65-69		0.93 (0.77-1.13)	1.02 (0.77-1.14)
70-74		1.21 (0.91-1.39)	1.14 (0.92-1.40)
75-79		1.11 (0.86-1.43)	1.10 (0.85-1.42)
80+		1.45 (1.14-1.83) **	1.44 (1.13-1.82) **
**Sex**			
Male		Ref	Ref
Female		0.75(0.64-0.88) ***	0.76 (0.65-0.90) **
**Education**			
No Schooling		Ref	Ref
Primary		1.46 (1.22-1.74) ***	1.50 (1.25-1.81) ***
Secondary		1.51 (1.21-1.88) ***	1.55 (1.23-1.94) ***
Higher Secondary		1.36 (0.92-2.01)	1.38 (0.91-2.05)
Graduate		1.15 (0.75-1.75)	1.11 (0.71-1.73)
Professional Couse		0.57 (0.17-0.80) ***	0.57 (0.21-0.83) **
**Religion**			
Hindu		Ref	Ref
Muslim		1.32 (1.22-1.74) **	1.35 (1.08-1.68) **
Christian		1.34 (1.02- 1.90) **	1.31 (1.04- 1.85) **
Other		0.73 (0.50-1.07)	0.64 (0.43- 0.96) **
**Caste**			
ST		Ref	Ref
SC		1.25 (1.06-1.63) *	1.09 (0.80-1.48)
OBC		0.58 (0.33-1.00) *	0.54 (0.31-0.95) **
Other		0.19 (0.02-1.36) *	0.21 (0.02-1.51)
Ever diagnosed with stroke			
No			
Yes		1.57 (1.12-2.21) **	1.57 (1.12-2.21) **
Ever diagnosed with hypertension			
No			
Yes		1.47 (1.26-1.72) ***	1.49 (1.27-1.74) ***
Ever diagnosed with cancer			
No			
Yes		1.35 (0.68-2.68)	1.37 (0.69-2.73)
**Diabetic**			
No		Ref	Ref
Yes		1.14 (0.94-1.38)	1.20 (1.01-1.46) *
**Habit Of Alcohol**			
No		Ref	Ref
Yes		0.66 (0.49-0.95) **	0.70 (0.52-0.95) **
**Place of Residence**			
Rural			Ref
Urban			0.94 (0.79-1.12)
**Region**			
South			Ref
Central			1.49 (1.14-1.95) **
Eastern			1.51 (1.20-1.91) ***
Western			1.15 (0.86-1.54)
Northern			1.50 (1.18-1.91) ***
North East			1.65 (1.22-2.24) ***
**Income**			
< 1 Lacs			Ref
1 - 1.5 Lacs			0.99 (0.80-1.24)
1.5-2 Lacs			0.86 (0.66-1.13)
2-3 Lacs			0.91 (0.80-1.32) ***
> 3 Lacs			0.95 (0.78-1.17) ***
**Toilet Facility**			
Flush Or Pour Flush Toilet			Ref
Pit Latrine			0.76 (0.63-0.93) **
Twin Pit/Composting Toilet			0.82 (0.53-1.26)
No Facility, Use Open Space			0.91 (0.73-1.15)

Ref = reference group, *** = p < 0.001, and ** = p < 0.05, * = p < 0.10. AOR = Adjusted odds ratios for the outcome variable, 95% confidence intervals.

Age emerged as a significant predictor, with the odds of UTIs increasing with advancing age. Elderly individuals aged 80 years or older were 45% more likely to have UTIs compared than those aged 60–64 years. Women were at lower risk, with a 25% lower likelihood of UTIs compared to men (AOR: 0.75, 95% CI: 0.64-0.88). Interestingly, higher education levels were associated with increased UTI prevalence; however, those who completed professional courses had a 43% lower likelihood of UTIs (AOR: 0.57, 95% CI: 0.17–0.80). Religion also played a role, with Muslims and Christians being 32% and 34% more likely, respectively, to experience UTIs compared to Hindus.The social category was not a significant predictor in any model.

In Model 3, elderly individuals with UTIs were 20% more likely to be diabetic (AOR: 1.20, 95% CI: 1.01–1.64). Those who experienced stroke and hypertension were 57% and 49% more likely to have UTIs. Further, individuals diagnosed with cancer were 37% more likely to report UTIs; however, this association was not statistically significant. Place of residence was found to be an insignificant factor. Regionally, individuals with UTIs were 49%, 50%, and 65% more likely to be from Central, Northern, and North-eastern India, respectively, compared to Southern India. Habitual alcohol consumption was associated with a 30% lower likelihood of experiencing UTIs (AOR: 0.70; 95% CI: 0.51–0.95). Increasing annual income was associated with a decline in UTI prevalence, with elderly individuals earning more than ₹3,00,000 annually being 18% less likely to have UTIs (AOR: 0.82, 95% CI: 0.78–0.90). Additionally, elderly individuals using pit latrines were 25% less likely to have UTIs compared to those using flush or pour-flush toilets (AOR: 0.75, 95% CI: 0.62–0.92).

## Discussion

The present study explores the association between the prevalence of UTIs and anemia among the elderly population in India. The findings uncovered a strong association, with elderly individuals experiencing UTIs  nearly five times more likely to be anemic. The prevalence of UTIs among the elderly was found to be at 2.5%. The prevalence rates of UTIs may be influenced by factors such as early diagnosis and awareness of the condition. In high-income countries, greater awareness and routine health screenings often result in higher documented prevalence rates. In contrast, low- and middle-income countries, including India, face systemic challenges such as limited healthcare access and reduced awareness, which may contribute to underdiagnosis and subsequently lower observed prevalence among the elderly.

The study identified a strong association between UTIs and anemia, corroborating findings from previous research [17,18,16,35]. It is important to acknowledge that these earlier studies primarily focused on pregnant women, indicating a need for further clinical research to investigate the underlying relationship between UTIs and anemia in the elderly population. Another objective of the study was to examine the determinants of UTIs among the elderly in India. Our findings revealed that individuals over 80had a higher risk of developing UTIs. This result aligns with previous studies conducted in Scandinavian countries [38], Saudi Arabia [43], and Pennsylvania [49]. The heightened vulnerability of older adults to UTIs can be attributed to age-related physiological changes, sex-based differences, typical symptom presentation, and diagnostic challenges. Although dipstick urinalysis is a practical diagnostic tool, its accuracy may be compromised, particularly when symptoms overlap with other conditions, further increasing the risk for the elderly [50]. Older men were more susceptible to UTIs than women in our study. This finding is in contrast with previous research [38,39,51].

An interesting finding of our study was that a higher educational level was associated with a higher likelihood of self-reported UTIs among the elderly. However, this likelihood decreased when the elderly individuals had completed any professional courses, which may be because educated individuals are more aware of such infections and are thus more likely to report them. Elderly individuals with professional courses may possess greater knowledge or access to resources encouraging them to take preventive measures, reducing the likelihood of experiencing UTIs. This finding aligns with results from a quasi-experimental study conducted in Iran, which demonstrated that education enhances preventive practices for UTIs, supporting the role of knowledge in managing and preventing infections [52]. Elderly individuals practicing Christianity and Islam were at higher risk of UTIs, with a 26% and 37% increased likelihood, respectively, whichmay be due to a greater focus on traditional spirituality and less emphasis on health practices, similar to findings in the previous study [53]. Elderly individuals who reported having UTIs were more likely to have diabetes, which aligns with findings from several previous studies [28,30,16,54]. One potential explanation for this association is that a surge of glucose levels in the urine creates an environment conducive to bacterial growth, increasing the risk of UTIs. Additionally, diabetic neuropathy can impair bladder function, leading to urine retention and increasing the risk of UTIs [54, 55]. Previous studies have revealed that the experience of stroke is associated with an increased risk of UTIs among older adults; our study also had similar findings, where people who experienced stroke once were more likely to have UTIs [56–58]. Stroke can impair bladder control and mobility, increasing the risk of urinary retention or incontinence, which in turn makes older adults more susceptible to UTIs [57]

Annual income emerged as another significant predictor of UTIs among the elderly. An inverse association was observed, wherein the risk of UTIs declined with increasing income levels, which is in line with earlier studies [30,16,43]. This inverse relationship may be explained by improved access to healthcare, greater awareness, and better knowledge of preventive measures among higher-income individuals. A study from Nigeria further supports this association, suggestingthat income is linked toincreased pathogen presence in urine, mainly due to factors such as inadequate housing and poor sanitation [59]. Elderlies in north-eastern India were found to have a 70% increased risk of UTIs compared to those in South India. These differences may not be linked to a single factor but may be influenced by multiple factors, including healthcare accessibility, awareness programs, dietary, lifestyle habits, and access to clean water and sanitation. Micro-level studies in South India reported a prevalence of around 38% [60], while a study in north-eastern India found the prevalence to be as high as three-fifths [25]. However, further research is necessary to fully understand the underlying causes of these regional variations. To our surprise, the risk of UTIs was lower among elderlies who lacked toilet facilities or practiced open defecation. This finding contradicts existing literature [15]. and was statistically significant, thereby necessitating closer scrutiny.

The Indian government has implemented several initiatives to reduce the risk of UTIs, including the National Guidelines for Infection Prevention and Control in Healthcare Facilities, which outline the types of UTIs, associated risk factors, and preventive measures [61]. Beyond these national strategies, community-based screening programs should be integrated into the National programme for Health Care of the Elderly (NPHCE), including dual screening for UTIs and anemia and focusing on high-risk groups. Enhanced sanitation efforts under the Swachh Bharat Mission should be further strengthened, particularly in Eastern India, where prevalence appears more pronounced. Nonetheless, the effectiveness of these interventions at the grassroots level remains insufficiently understood, and subsequentresearch should assess their real-world impact. Environmental determinants also warrant greater attention through targeted improvements in sanitation infrastructure and access to safe drinking water, especially in rural and underserved areas where the burden is likely higher.

### Limitations

It is necessary that we acknowledge the limitations of this study. The cross-sectional design restricts the ability to establish a causal relationship between anemia and UTIs, and the possibility of a bidirectional association cannot be ruled out. While most existing evidence on this relationship comes from studies among pregnant women, mechanisms such as reduced immunity due to anemia or inflammation resulting from recurrent UTIs may also be relevant in older populations. Secondly, potential confounding variables that could independently influence both conditions were not fully accounted for, including medication use (e.g., antibiotics, NSAIDs, diuretics), nutritional deficiencies (iron, folate, vitamin B12), and hydration-related factors.Other factors could also be involved ,including immunological deterioration linked to frailty, urinary incontinence, and hormonal changes in older women. However, these variables were unavailable in the LASI dataset and could not be included in the analysis. The reliance on self-reported data introduces the possibility of recall or reporting bias, and the absence of laboratory-confirmed diagnostic measures limits clinical precision. These limitations reiterate the significance of future longitudinal studies that involveclinical validation and a wider set of health and behavioral determinants, as well as the necessity of interpreting the results with caution.

### Policy recommendations

Given the envisioned demographic shift towards an ageing population, these novel findings on anemia-UTI associations among Indian elderly populations demand strategic integration into India’s larger healthcare setup. Primary care protocols for older adults should include routine dual screening for UTIs and anemia, especially as part of the NPHCE. The study’s high-risk groups—males, individuals over 80, those with diabetes, hypertension, and patients already who are diagnosed with hypertension and anemic should be prioritised within this framework. To support early identification and intervention, frontline health workers particularly ASHAs and ANMs require specialised training to recognise overlapping symptoms and risk factors. For effective implementation, the NPHCE should be expanded to incorporate such training, while a tiered medication subsidy programme targeting low-income elderly citizens should be introduced to address the economic constraints identified in the study. Integrating these recommendations with initiatives like Anemia Mukt Bharat and the Health and Wellness Centres under Ayushman Bharat would strengthen care continuity. Establishing a monitoring mechanism to track reductions in UTI and anemia prevalence.

## Conclusion

The present research yields several important findings. Firstly, it revealed that the prevalence of UTIs among the elderly in India was 2.5%. Secondly, a strong association between UTIs and anemia was observed, although further clinical research is needed to understand the mechanisms underlying this relationship. The study also found that the risk of UTIs increases with advancing age and decreases with higher annual income. Higher likelihood of UTIs was observed among older adults practicing Islam or Christianity, residents of North-Eastern India, individuals with diabetes, hypertension, or a history of stroke. Moreover, older men were more likely than younger men to experience a UTI. Infections like UTIs among the elderly must be dealt with in view of India's changing demographics, as they can affect longevity and quality of life. As this study is cross-sectional and non-clinical, some limitations could be addressed via longitudinal and clinical studies. Future research should explore these aspects further.
