# Correction: Association between urinary tract infections and anemia among the elderly in India: Insights from the longitudinal aging study

**DOI:** 10.1371/journal.pgph.0005239

**Published:** 2025-09-24

**Authors:** Dilwar Hussain, Jenica Barnwal

The Data Availability statement for this article is incorrect. The correct statement is: This study used data from the Longitudinal Aging Study in India (LASI) Wave 1, conducted in 2017–18. The dataset is publicly available upon request from the International Institute for Population Sciences (IIPS), Mumbai, India. Access information is available at: https://www.iipsindia.ac.in/content/LASI-data.

There are a number of errors for Tables 1–3 from the article. Please see the correct Tables 1–3 here.

**Table 1:** Sample distribution of the study population by the background characteristics, India (LASI, 2017–18).

Background Characteristics	Sample (n)	Percent
Had UTIs		
No	30,020	97.45
Yes	784	2.55
Anemic		
Yes	1,230	3.99
No	29,574	96.01
Age (years)		
60-64	9,938	32.26
65-69	8,673	28.16
70-74	5,601	18.18
75-79	3,278	10.64
80+	3,314	10.76
Sex		
Male	14,793	48.02
Female	16,011	51.98
Place of Residence		
Rural	20,430	66.32
Urban	10,374	33.68
Education		
No schooling	16,566	53.78
Primary	7,378	23.95
Secondary	4,511	14.64
Higher secondary	1,064	3.45
Graduate	1,056	3.43
Professional course	229	0.74
Annual Income (lakhs)		
< 1	12,896	41.86
1-1.5	4,640	15.06
1.5-2	3,237	10.51
2-3	4,083	13.25
> 3	5,948	19.31
Region		
Southern	7,977	25.9
Central	4,194	13.62
Eastern	5,724	18.58
Western	3,475	11.28
Northern	5,707	18.53
North-eastern	3,727	12.1
Caste		
Scheduled tribe	5,082	16.5
Scheduled caste	5,109	16.59
Other backward class	11,666	37.87
Other	8,947	29.04
Religion		
Hindu	22,544	73.19
Muslim	3,642	11.82
Christian	3,089	10.03
Other	1,529	4.96
Ever diagnosed with stroke		
No	29,978	97.33
Yes	824	2.67
Ever diagnosed with hypertension		
No	20,046	65.07
Yes	10,758	34.93
Ever diagnosed with cancer		
No	30,569	99.25
Yes	235	0.75
Diabetic		
Yes	4,744	15.4
No	26,060	84.61
Habit of alcohol		
Yes	2,828	9.18
No	27,976	90.82
Toilet facility		
Flush or pour flush toilet	15,818	51.35
Pit latrine	7,554	24.52
Twin pit/composting toilet	1,013	3.29
No facility, use open space	6,419	20.83

**Table 2:** Prevalence of UTIs among the elderly across different background characteristics, India (LASI, 2017–18).

Background Characteristics	Prevalence of UTIs	P Value
Anemic		<0.001
Yes	10.83	
No	2.19	
Age (years)		<0.001
60-64	2.21	
65-69	2.41	
70-74	2.56	
75-79	3.05	
80+	3.58	
Sex		<0.001
Male	3.1	
Female	2.11	
Place of Residence		<0.001
Rural	2.51	
Urban	2.80	
Education		<0.001
No schooling	2.08	
Primary	3.37	
Secondary	3.06	
Higher secondary	2.86	
Graduate	3.72	
Professional course	2.34	
Annual Income (lakhs)		<0.001
< 1	2.38	
1-1.5	2.95	
1.5-2	2.95	
2-3	2.87	
> 3	2.31	
Region		<0.001
Southern	1.56	
Central	2.94	
Eastern	3.13	
Western	2.73	
Northern	2.49	
North-eastern	2.56	
Caste		<0.001
Scheduled tribe	2.0	
Scheduled caste	2.59	
Other backward class	2.31	
Other	3.2	
Religion		<0.001
Hindu	2.6	
Muslim	2.92	
Christian	1.52	
Other	1.96	
Ever diagnosed with stroke		<0.001
No	2.53	
Yes	4.45	
Ever diagnosed with hypertension		<0.001
No	2.05	
Yes	3.7	
Ever diagnosed with cancer		0.195
No	2.59	
Yes	1.75	
Diabetic		<0.001
Yes	3.1	
No	2.5	
Habit of alcohol		<0.001
Yes	2.64	
No	1.88	
Toilet facility		<0.001
Flush or pour flush toilet	2.56	
Pit latrine	2.87	
Twin pit/composting toilet	3.18	
No facility, use open space	2.35	

**Table 3:** Logistic regression results showing the influence of anemia and other covariates on UTIs among the elderly, India (LASI, 2017–18).

Background characteristics	Model 1	Model 2	Model 3
Anemic			
No	Ref	Ref	Ref
Yes	5.49 (4.52-6.67) ***	5.95 (4.87-7.27) ***	5.87 (4.80-7.18) ***
Age (years)			
60-64		Ref	Ref
65-69		0.93 (0.77-1.13)	1.02 (0.77-1.14)
70-74		1.21 (0.91-1.39)	1.14 (0.92-1.40)
75-79		1.11 (0.86-1.43)	1.10 (0.85-1.42)
80+		1.45 (1.14-1.83) **	1.44 (1.13-1.82) **
Sex			
Male		Ref	Ref
Female		0.75(0.64-0.88) ***	0.76 (0.65-0.90)**
Education			
No schooling		Ref	Ref
Primary		1.46 (1.22-1.74) ***	1.50 (1.25-1.81) ***
Secondary		1.51 (1.21-1.88) ***	1.55 (1.23-1.94) ***
Higher secondary		1.36 (0.92-2.01)	1.38 (0.91-2.05)
Graduate		1.15 (0.75-1.75)	1.11 (0.71-1.73)
Professional course		0.57 (0.17-0.80) ***	0.57 (0.21-0.83) **
Religion			
Hindu		Ref	Ref
Muslim		1.32 (1.22-1.74) **	1.35 (1.08-1.68) **
Christian		1.34 (1.02- 1.90) **	1.31 (1.04- 1.85) **
Other		0.73 (0.50-1.07)	0.64 (0.43- 0.96) **
Caste			
Scheduled tribe		Ref	Ref
Scheduled caste		1.25 (1.06-1.63)*	1.09 (0.80-1.48)
Other backward class		0.58 (0.33-1.00)*	0.54 (0.31-0.95)**
Other		0.19 (0.02-1.36) *	0.21 (0.02-1.51)
Ever diagnosed with stroke			
No			
Yes		1.57 (1.12-2.21)**	1.57 (1.12-2.21)**
Ever diagnosed with hypertension			
No			
Yes		1.47 (1.26-1.72)***	1.49 (1.27-1.74)***
Ever diagnosed with cancer			
No			
Yes		1.35 (0.68-2.68)	1.37 (0.69-2.73)
Diabetic			
No		Ref	Ref
Yes		1.14 (0.94-1.38)	1.20 (1.01-1.46) *
Habit of alcohol			
No		Ref	Ref
Yes		0.66 (0.49-0.95)**	0.70 (0.52-0.95)**
Place of Residence			
Rural			Ref
Urban			0.94 (0.79-1.12)
Region			
South			Ref
Central			1.49 (1.14-1.95) **
Eastern			1.51 (1.20-1.91) ***
Western			1.15 (0.86-1.54)
Northern			1.50 (1.18-1.91) ***
North east			1.65 (1.22-2.24) ***
Annual Income (lakhs)			
< 1			Ref
1-1.5			0.99 (0.80-1.24)
1.5-2			0.86 (0.66-1.13)
2-3			0.91 (0.80-1.32) ***
> 3			0.95 (0.78-1.17) ***
Toilet facility			
Flush or pour flush toilet			Ref
Pit latrine			0.76 (0.63-0.93) **
Twin pit/composting toilet			0.82 (0.53-1.26)
No facility, use open space			0.91 (0.73-1.15)

Note: Ref = reference group, *** = p < 0.001, and ** = p < 0.05, * = p < 0.10. AOR = Adjusted odds ratios for the outcome variable, 95% confidence intervals.
